# Diagnostic Performance of a Smartphone‐Based Photoplethysmographic Application for Atrial Fibrillation Screening in a Primary Care Setting

**DOI:** 10.1161/JAHA.116.003428

**Published:** 2016-07-21

**Authors:** Pak‐Hei Chan, Chun‐Ka Wong, Yukkee C. Poh, Louise Pun, Wangie Wan‐Chiu Leung, Yu‐Fai Wong, Michelle Man‐Ying Wong, Ming‐Zher Poh, Daniel Wai‐Sing Chu, Chung‐Wah Siu

**Affiliations:** ^1^ Cardiology DivisionDepartment of MedicineLi Ka Shing Faculty of MedicineThe University of Hong KongHong KongChina; ^2^ Cardiio Inc.CambridgeMA; ^3^ Department of Family Medicine and Primary HealthcareHong Kong East ClusterHong KongChina

**Keywords:** atrial fibrillation, mobile app, photoplethysmography, screening, Atrial Fibrillation

## Abstract

**Background:**

Diagnosing atrial fibrillation (AF) before ischemic stroke occurs is a priority for stroke prevention in AF. Smartphone camera–based photoplethysmographic (PPG) pulse waveform measurement discriminates between different heart rhythms, but its ability to diagnose AF in real‐world situations has not been adequately investigated. We sought to assess the diagnostic performance of a standalone smartphone PPG application, Cardiio Rhythm, for AF screening in primary care setting.

**Methods and Results:**

Patients with hypertension, with diabetes mellitus, and/or aged ≥65 years were recruited. A single‐lead ECG was recorded by using the AliveCor heart monitor with tracings reviewed subsequently by 2 cardiologists to provide the reference standard. PPG measurements were performed by using the Cardiio Rhythm smartphone application. AF was diagnosed in 28 (2.76%) of 1013 participants. The diagnostic sensitivity of the Cardiio Rhythm for AF detection was 92.9% (95% CI] 77–99%) and was higher than that of the AliveCor automated algorithm (71.4% [95% CI 51–87%]). The specificities of Cardiio Rhythm and the AliveCor automated algorithm were comparable (97.7% [95% CI: 97–99%] versus 99.4% [95% CI 99–100%]). The positive predictive value of the Cardiio Rhythm was lower than that of the AliveCor automated algorithm (53.1% [95% CI 38–67%] versus 76.9% [95% CI 56–91%]); both had a very high negative predictive value (99.8% [95% CI 99–100%] versus 99.2% [95% CI 98–100%]).

**Conclusions:**

The Cardiio Rhythm smartphone PPG application provides an accurate and reliable means to detect AF in patients at risk of developing AF and has the potential to enable population‐based screening for AF.

## Introduction

Atrial fibrillation (AF), the most common sustained cardiac arrhythmia encountered in clinical practice,^1^, ^2^ confers a 5‐fold higher risk of ischemic stroke^2^ and is asymptomatic in at least one‐third of patients.^3^ Further, AF‐related strokes are more severe and more often disabling or fatal compared with strokes from other causes.^4^ Although long‐term oral anticoagulation therapy effectively prevents about two‐thirds of ischemic strokes among patients with AF,^5^ nearly 25% of patients with stroke or transient ischemic attack have AF diagnosed only after the event,^6^ precluding them from any meaningful primary preventive therapy. Thus, the diagnosis of AF before the occurrence of ischemic stroke is recognized as an integral component of successful stroke prevention.

The European Society of Cardiology advocates pulse palpation, followed by an electrocardiogram (ECG) if the pulse is irregular, as opportunistic screening for AF in patients aged ≥65 years.^1^, ^7^ Nonetheless, opportunistic screening is not routinely performed in many primary care settings because of the time‐consuming nature of routine pulse palpation and subsequent ECG measurement. Given that AF episodes can be brief and infrequent, reliance on a single spot‐check at a clinic is likely to result in a missed diagnosis in many patients with paroxysmal AF. The recent STROKESTOP study showed that intermittent short ECG recordings at home repeated over a longer‐term period produced significantly better sensitivity for AF detection, with 4 times as many cases diagnosed compared with a single time‐point measurement.^8^ Thus, mobile devices that are capable of detecting AF and that can be operated regularly by patients at home may be able to bridge this gap in clinical practice. Such mobile devices may also provide AF patients with an important tool for self‐management of their condition, especially for those with paroxysmal AF.

Mobile devices and applications are profoundly transforming the practice of medicine and the way health decisions are made. Smartphones can now act as ECG monitors by interfacing with peripherals such as a special smartphone case with embedded electrodes to acquire, store, and transfer single‐channel ECG rhythms.^9^ A successful example is the AliveCor Heart Monitor (AliveCor Inc), which has already been US Food and Drug Administration cleared and Conformité Européenne (CE) marked. Photoplethysmography (PPG), an optical method that measures changes in tissue blood volume caused by the pressure pulse, has also been shown to be possible using a smartphone without any additional peripherals.^10^, ^11^ The PPG waveform can be acquired using a smartphone camera to measure pulsatile changes in light intensity reflected from a finger illuminated by the pseudo‐white LED smartphone flash and placed in contact with the camera.^11^, ^12^ Although others have recently reported the feasibility of using smartphone PPG to detect AF in a group of patients preselected for their heart rhythm status,^13^, ^14^ they did not demonstrate the ability to diagnose and/or screen AF in ambulatory outpatients.^15^ Therefore, the performance of smartphone PPG for AF screening in real‐world situations where various other arrhythmias are common remains unclear.

The primary aim of this study was to assess the diagnostic performance of a standalone smartphone PPG application, Cardiio Rhythm,^16^ for AF screening in a primary care setting.

## Methods

### Study Design

This prospective screening study was coordinated by the University of Hong Kong and the Department of Family Medicine and Primary Healthcare Service, Hong Kong East Cluster, Hospital Authority, Hong Kong. The study protocol was approved by the local institutional review board. Patients were recruited from Chai Wan General Outpatient Clinic in Hong Kong from May through June 2015. Patients were eligible if they had a history of hypertension and/or diabetes mellitus or were ≥65 years of age. Patients with a pacemaker or implantable defibrillator were excluded from the study. Informed consent was obtained from all patients who fulfilled the inclusion criteria.

### Screening Procedure

**Figure 1:**
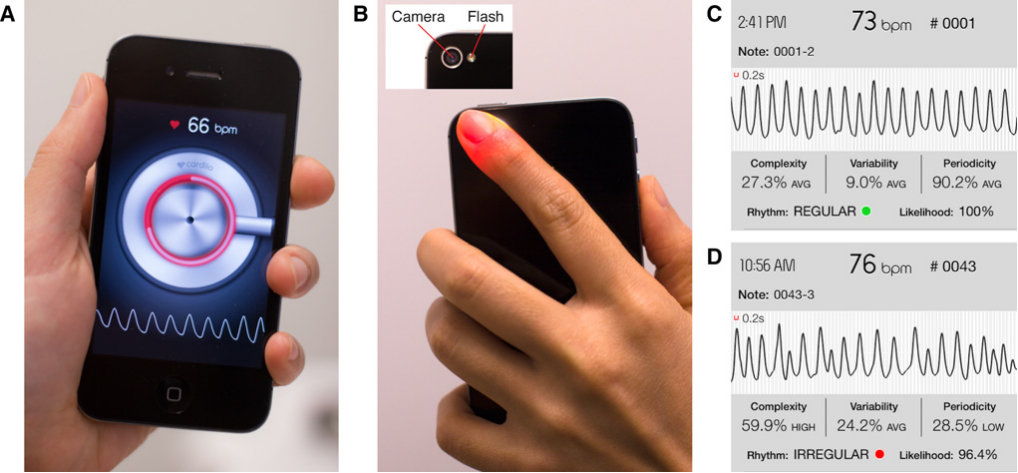
Smartphone camera‐based photoplethysmography (PPG) measurements of the pulse waveform. A, The Cardiio Rhythm standalone smartphone application. B, A finger is placed in contact with the smartphone camera and is illuminated by the adjacent LED flash. Examples of PPG recordings from a patient in (C) sinus rhythm and a patient in (D) atrial fibrillation (Video S1).

A bipolar lead I ECG recording was first obtained from all patients using an AliveCor Heart Monitor (1st generation; AliveCor Inc). The AliveCor Heart Monitor is Food and Drug Administration cleared, CE marked, and clinically validated for the recording of single‐channel lead I ECGs.^17^, ^18^ For each patient, a single‐lead ECG tracing was acquired for 30 seconds with placement of ≥2 fingers from each hand on the device electrodes. The ECG recordings were transmitted to an iPad mini (Apple Inc installed with the AliveECG application (version 2.2.2) that interpreted the ECGs with an automated algorithm. For patients whose ECG tracings were initially affected by artifacts, they were instructed by the trained observers to repeat the recording so as to provide optimal tracing for subsequent reading by cardiologists. Immediately following completion of the ECG recording, 3 PPG waveforms were acquired sequentially from each patient using an iPhone 4S (Apple Inc) running the Cardiio Rhythm smartphone application (Cardiio Inc). PPG waveform recordings were performed by the patients under the supervision of trained observers. Patients were instructed to place the tip of their index finger of either hand on the camera of the iPhone (Figure 1, Video S1). Each PPG waveform recording lasted 17.1 seconds and was classified automatically by the Cardiio Rhythm smartphone application as “Regular” or “Irregular.” A diagnosis of AF was produced if at least 2 of 3 PPG waveform recordings from a single patient were classified as “Irregular.” When a diagnosis of AF was made by the Cardiio Rhythm application, the AliveCor automated AF detection algorithm, or both, a full 12‐lead ECG was performed within 15 minutes of the initial screening. An independent individual printed out the AliveCor ECG tracing with the automated rhythm interpretation redacted. Finally, 2 cardiologists who were blinded to the Cardiio Rhythm classifications, AliveCor automated interpretations, and patient baseline information independently reviewed the single‐lead ECG printouts to provide a reference diagnosis by using standard criteria.^19^

### Cardiio Rhythm Smartphone Application

PPG waveforms were acquired using the iPhone's LED flash to illuminate a patient's finger, after which the iPhone camera captured the reflected light that changes according to the arterial blood volume pulsations. PPG waveforms were sampled at 30 Hz, and each measurement represented 512 samples (ie, 17.1 seconds). PPG waveforms were filtered by using a bandpass filter (0.7–4.0 Hz) to remove baseline wander and high‐frequency noise. The approach for detecting the presence of AF was based on a lack of repeating patterns in the PPG waveform because of the irregular rhythm of AF.^16^ This was achieved by using a Support Vector Machine to classify each PPG waveform as AF or non‐AF based on the self‐similarity of the waveform. Posterior class probabilities were computed by approximation using a sigmoid function.

### Rhythm Diagnosis

The primary analysis was to evaluate the diagnostic accuracy of the Cardiio Rhythm smartphone application to detect AF against a reference diagnosis made following interpretation of a single‐lead ECG by 2 blinded and independent cardiologists. The Cardiio Rhythm smartphone application produced a diagnosis of AF if at least 2 of 3 pulse waveform recordings from a single patient were classified as “Irregular.” Otherwise, the patient was classified as non‐AF. For comparison, we also evaluated the AliveCor automated AF detection algorithm built into the AliveECG application against the reference standard from the 2 cardiologists. A diagnosis of AF was made for the AliveCor's algorithm if the AliveECG application displayed “Possible AF.” Otherwise, the patient was classified as non‐AF.

### Statistical Analysis

Continuous and discrete variables are expressed as mean±SD and percentages, respectively. Sensitivity, specificity, likelihood ratio, and predictive value for AF diagnosis were calculated as simple proportions with corresponding 95% CI for the Cardiio Rhythm smartphone application and the AliveCor automated algorithm. To examine the possible improvement (or deterioration) of the Cardiio Rhythm smartphone application over the AliveCor automated AF detection algorithm, the net reclassification improvement (NRI) was calculated by using the following formula:

$$\text{NRI}=\left(\frac{\text{AF correctly reclassified by Cardiio}-\;\text{AF incorrectly reclassified by Cardiio}}{\text{number of AF}}\right)+\left(\frac{\text{Non‐AF correctly reclassified by Cardiio}-\text{Non‐AF incorrectly reclassified by Cardiio}}{\text{Number of non‐AF}}\right)$$

A positive NRI indicates improvement of the Cardiio Rhythm smartphone application over AliveCor automated AF detector in AF detection. Calculations were performed by using SPSS software version 21.0 (IBM Corp, USA) and MedCalc version 13.1.2 (MedCalc Software, Belgium).

## Results

**Figure 2:**
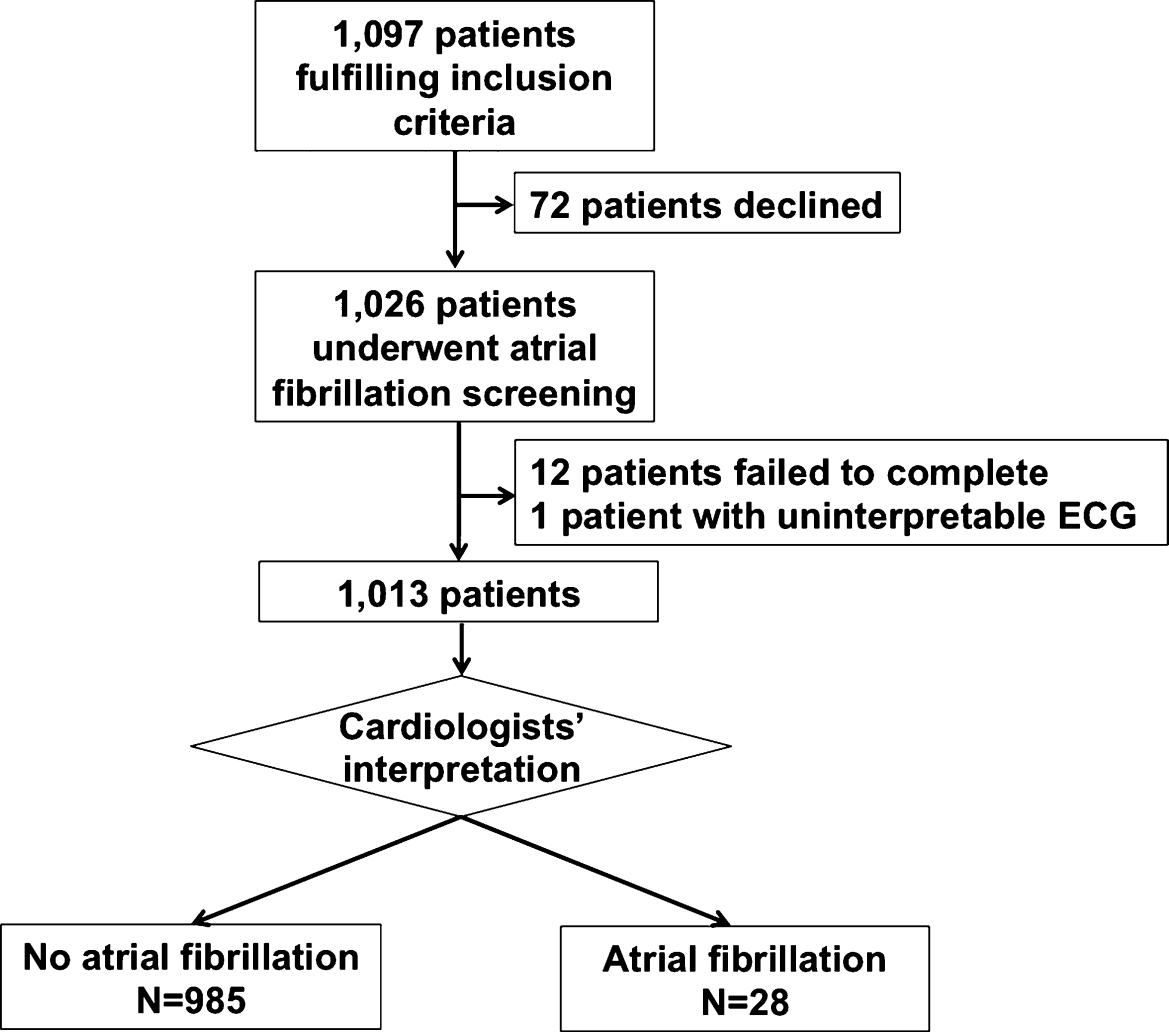
Study enrollment and flow.

Between May and June 2015, 1098 patients who fulfilled the inclusion criteria of the present study were invited to participate in the AF screening study; 72 (6.5%) declined. Of the consenting patients, 12 were excluded from the final analysis because of failure to complete the screening process, and 1 patient was excluded because the ECG tracings were uninterpretable by the cardiologists. As a result, 1013 patients were included in this study (Figure 2). Table 1 summarizes the characteristics of the study population. The mean age was 68.4±12.2 years; 474 (46.8%) patients were male. Hypertension was present in 916 (90.4%) patients, and diabetes mellitus was present in 371 (36.6%). In addition, there were 164 (16.2%) patients with coronary artery disease and 106 (10.5%) patients with a history of previous stroke. The mean CHA_2_DS_2_‐VASc score was 3.0±1.5.

**Table 1:** Demographics of Study Population

Characteristics	Number (%) (N=1013)
Age, mean±SD, y	68.4±12.2
Male	474 (46.8)
Hypertension	916 (90.4)
Diabetes mellitus	371 (36.6)
Coronary artery disease	164 (16.2)
Previous myocardial infarction	33 (3.3)
Heart failure	45 (4.4)
Previous stroke	106 (10.5)
CHA_2_DS_2_‐VASc score	3.0±1.5

CHA_2_DS_2_‐VASc score: congestive heart failure=1 point; hypertension=1 point; age ≥75 years=1 point and age=65 to 74 years=1 point; diabetes mellitus=1 point; previous stroke=2 points; va: vascular disease=point; sex category (female)=1 point.

**Figure 3:**
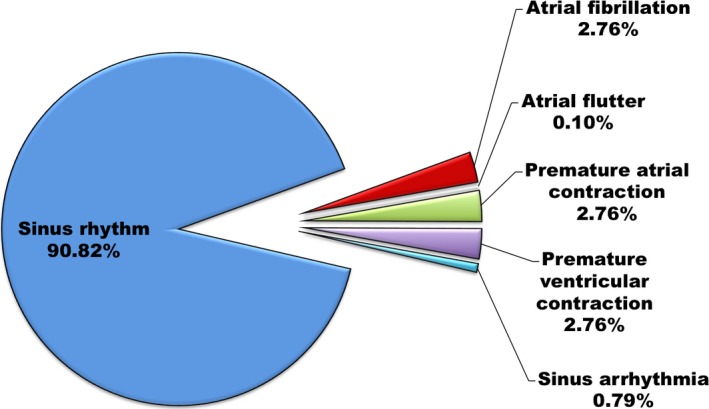
Rhythm diagnoses of the study population based on interpretation by 2 independent cardiologists of a 30‐second bipolar lead I ECG.

Of these 1013 patients, 920 (90.82%) were deemed to be in sinus rhythm based on the 2 cardiologists’ interpretation of the single‐lead ECG recording (Figure 3). AF was diagnosed in 28 (2.76%) patients and confirmed with a standard 12‐lead ECG. Among these 28 patients, 23 patients had a prior history of AF (all patients had either persistent or permanent AF documented); therefore, 5 (17.9%) of the 28 patients had newly diagnosed AF detected with the screening test. Other abnormal non‐AF rhythms detected in the study population included atrial flutter (n=1, 0.1%), premature atrial contractions (n=28, 2.76%), premature ventricular contractions (n=28, 2.76%), and sinus arrhythmias (n=8, 0.79%).

**Figure 4:**
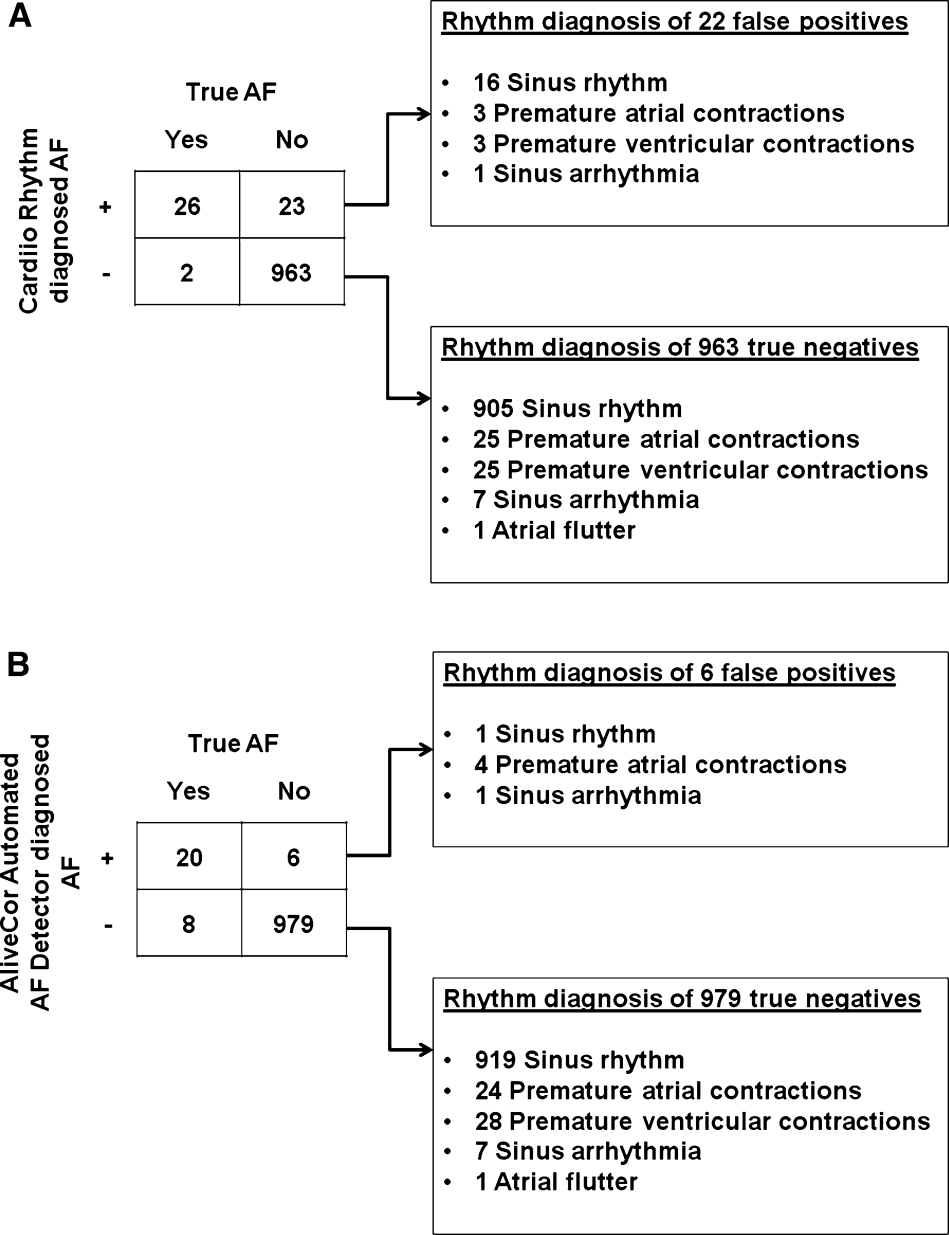
Contingency tables for atrial fibrillation detection and rhythm diagnoses of (A) the Cardiio Rhythm smartphone application and (B) the AliveCor Automated AF detector.

Of the 28 patients confirmed to have AF, 18 (64%) patients were found to be positive for AF by both the Cardiio Rhythm smartphone application and the AliveCor automated AF detector, 8 (29%) patients were classified by the Cardiio Rhythm smartphone application as AF alone, and 2 (7%) patients were classified as AF by the AliveCor automated AF detector alone. The Cardiio Rhythm smartphone application correctly identified AF in 26 of 28 AF patients and produced 23 false‐positive results. Figure 4 depicts the contingency table and rhythm diagnosis of the Cardiio Rhythm smartphone application. Among the 23 patients with a false‐positive result by the Cardiio Rhythm smartphone application, 16 were in sinus rhythm (69.6%), 3 had premature atrial contractions (13%), 3 had premature ventricular contractions (13%), and 1 had sinus arrhythmia (4.3%) (Figure 4A). The PPG waveform of the false‐positive results from patients who were in sinus rhythm contained motion and noise artifacts. The corresponding sensitivity and specificity of the Cardiio Rhythm smartphone application for AF detection were 92.9% (95% CI 76.5–99.1%) and 97.7% (95% CI 96.5–98.5%), respectively (κ=0.67). In this population of patients with an AF prevalence of 2.76%, the positive predictive value of the Cardiio Rhythm smartphone application for AF detection was 53.6% (95% CI 38.3–67.5%) and the negative predictive value was 99.8% (95% CI 99.3–100.0%). The positive likelihood ratio was 39.8 (95% CI 26.2–60.3), and the negative likelihood ratio was 0.07 (95% CI 0.02–0.28).

The Cardiio Rhythm smartphone application was also able to classify 904 (98.3%) of 920 patients in sinus rhythm, 25 (89.3%) of 28 patients with premature atrial contractions, 25 (89.3%) of 28 patients with premature ventricular contractions, 7 of 8 patients with sinus arrhythmia, and 1 of 1 patient in atrial flutter correctly as non‐AF patients (Table 2).

**Table 2:** Specificity of the Cardiio Rhythm Smartphone Application for AF Detection in Patients With Non‐AF Rhythm

Rhythm	No. of Patients	Specificity (%)
Premature atrial contraction	28	89.3
Premature ventricular contraction	28	89.3
Atrial flutter	1	100
Sinus arrhythmia	8	87.5
Sinus rhythm	920	98.3

The AliveCor automated AF detector detected AF in 20 of 28 AF patients (Figure 3), corresponding to a sensitivity of 71.4% (95% CI 51.3–86.8%), and produced 6 false positive results, corresponding to a specificity of 99.4% (95% CI 98.7–99.8%) (κ=0.73). Among the 6 false‐positive results, 1 was sinus rhythm, 4 were premature atrial contractions, and 1 was sinus arrhythmia. The positive predictive value of the AliveCor automated AF detector for AF was 77.2% (95% CI 56.1–91.4%) and the negative predictive value was 99.2% (95% CI 98.4–99.7%). The positive likelihood ratio was 117.3 (95% CI 51.1–269.3); the negative likelihood ratio was 0.29 (95% CI 0.16–0.52). The NRI between the Cardiio Rhythm smartphone application and AliveCor automated AF detector in AF detection was 0.198, indicating a net reclassification improvement of the former over the latter.

## Discussion

In this study, we evaluated the diagnostic performance of a smartphone‐based PPG application, Cardiio Rhythm, for AF screening in a real‐world primary healthcare setting. Our results showed that the Cardiio Rhythm smartphone application accurately identified patients with AF in the primary healthcare setting with a high sensitivity of 92.9% and a high specificity of 97.7%; this performance was comparable to that of an ECG‐based device, the AliveCor automated AF detector.

Previous work from McManus and colleagues demonstrated that analysis of smartphone PPG recordings can differentiate between AF and sinus rhythm without ectopy.^13^ Recently, the investigators showed that their method could also distinguish between AF and sinus rhythm with or without premature contractions.^14^ A drawback of these earlier studies is that the participants were preselected based on prior knowledge of their heart rhythm. In addition, the results from these highly preselected groups including patients undergoing cardioversion and inpatients on the cardiac telemetry unit cannot be extrapolated to a much wider patient population as in a screening setting. In our study, the participants were representative of those who may benefit the most from population screening for AF that should target high‐risk patients.^8^, ^20^ The prevalence of AF (2.76%) in this study was largely similar to previously reported series.^21^ Detection of AF among these individuals would change patient management because these patients were likely to be ideal candidates for anticoagulation based on their CHA_2_DS_2_‐VASc score.^1^, ^19^, ^22^ This would enable earlier treatment to maximize the overall benefit of AF screening.

In this real‐world setting for population‐based AF screening, the Cardiio Rhythm smartphone application showed a high sensitivity and high specificity. Considering that the positive predictive value is not a fixed characteristic of a test and is dependent on the prevalence of the disease in the population tested, it is not surprising that the positive predictive value of Cardiio Rhythm was relatively low in our study population that had a low prevalence of AF.^23^ The application was also fairly immune to other non‐AF arrhythmias such as premature atrial contractions, premature ventricular contractions, and sinus arrhythmia. The majority of false positives originated from pulse waveforms that were corrupted by finger movement artifacts that may have affected the detection algorithm. This issue may lead to a reduction in specificity when the smartphone application is used outside the clinic because of potentially more motion artifacts in an unsupervised setting, eg, using it repeatedly at home over a period of weeks or even longer. A high number of false positives could create additional work for clinicians to rule out AF. One way to overcome this limitation is to add a pulse waveform quality assessment step prior to running the AF detection algorithm to reject recordings that are corrupted or too noisy and prompt the user to retake a measurement. Another drawback of the application is the inability to detect atrial flutter that may also confer some risk of stroke and frequently accompanies AF.^24^, ^25^ The application also requires proper finger contact with the camera to obtain an accurate measurement; this might be less familiar, or even difficult, for some patients, particularly the elderly.

A surprising finding in this study was that the AliveCor ECG‐based automated AF detector achieved a relatively low sensitivity of 71.4% compared with previously published estimates by Lau et al and Lowres et al of 98% and 98.5%, respectively.^9^, ^18^ These earlier studies used an older version of the AliveECG app, whereas we used the most updated version at the time of study commencement, so it may be that AliveCor modified their automated algorithm between the different app versions. Moreover, a recent study by Desteghe et al reported a much lower sensitivity of 54.5% and 78.9% for AliveCor's automated algorithm in detecting AF among cardiology and geriatric patients,^26^ respectively, which is in agreement with our findings here.

The underlying mechanism leading to the 8 false negatives produced by AliveCor's automated algorithm in our study is unclear. Nonetheless, a benefit of using ECG‐based systems to screen for AF is having the option to overread the ECG tracings, which can help a clinician rule in or rule out AF. Currently, no such mechanism exists to overread PPG tracings.

One of the greatest advantages of a smartphone PPG application is that it does not require any additional hardware investment, making it more accessible and appealing to patients. The nature of smartphone PPG as a software‐based solution allows for broad screening eligibility for every smartphone owner. This is particularly attractive because of the highly accessible nature of these devices. In the United States, smartphone ownership among the elderly continues to increase rapidly, with 27% of people age ≥65 years and 54% of those aged 50 to 64 years already owning a smartphone.^27^ In addition, established distribution channels such as the Apple App Store or Google Play store can realize mass screening for AF. Future research is warranted to determine how well smartphone PPG performs when used by patients in an unsupervised home setting for self‐testing for AF. For example, a high‐risk cohort suitable for screening for AF could be enrolled and provided with an ambulatory ECG patch monitor to be worn for 2 weeks. Participants would be asked to use the Cardiio Rhythm smartphone application at least twice daily during the ECG monitoring period. The diagnostic accuracy of the smartphone application would then be evaluated against the reference ECG recordings.

A limitation of this study is that we did not record a formal 12‐lead ECG in every participant. Instead, we asked 2 cardiologists to independently overread each single‐lead ECG and provide a diagnosis. This was necessary given the time and cost constraints inherent in dealing with a large number of patients. The lead I ECG tracings, particularly those that use dynamic filtering and gain control, might dramatically risk the loss of P waves and create false‐negative results. We acknowledged that diagnostic uncertainty might result from poor‐quality tracings with motion artefact, low voltage of P waves in lead I, or where sinus arrhythmia and frequent atrial ectopics mimic AF. Nonetheless, all patients identified by the cardiologists to have AF received a follow‐up 12‐lead ECG for further confirmation of the diagnosis. In addition, there is a possibility of an underdiagnosis of atrial flutter given that atrial flutter is not usually apparent in lead I of an ECG. For example, in a case of atrial flutter with regular conduction and without flutter waves in lead I, neither the single‐lead ECG nor the PPG pulse waveform could be expected to provide a diagnosis. Last but not least, both PPG recordings using the Cardiio Rhythm smartphone application and single‐lead ECG recordings using AliveCor AF detector were performed under medical supervision in a primary healthcare setting. It remains unclear whether these applications would achieve the same accuracy in an unsupervised condition.

## Conclusion

The Cardiio Rhythm smartphone PPG application is able to detect AF with a high sensitivity and specificity that is comparable to the Food and Drug Administration–cleared, ECG‐based AliveCor automated AF detector. Nonetheless, its intended application is as a screening tool, and not as a substitute for the standard ECG and its interpretation by a cardiologist. For a screening test, it is important to have a high specificity and negative predictive value.^28^ False‐negative results are undesirable, but a moderate number of false‐positive results are acceptable given that all those positive to the screening test will be evaluated again (eg, with a full 12‐lead ECG for final diagnosis). Our results suggest that the high specificity and negative predictive value of the Cardiio Rhythm smartphone application, together with its low cost and broad accessibility, may make massive population‐wide AF screening highly feasible.

## Sources of Funding

None. Cardiio, Inc provided the Cardiio Rhythm smartphone application and iPhones for study purposes.

## Disclosures

Drs Yukkee Poh and Ming‐Zher Poh are employees of Cardiio, Inc and have an ownership stake in the company. Dr Ming‐Zher Poh has a patent for the AF detection algorithm described in the article. There are no other potential conflicts of interest relevant to this study.

## Supporting information


**Video S1.** This video demonstrates how the photoplethysmographic recordings were obtained from a patient in sinus rhythm, followed by a patient in atrial fibrillation.
